# [Retracted] N6-methyladenosine upregulates miR-181d-5p in exosomes derived from cancer-associated fibroblasts to inhibit 5-FU sensitivity by targeting NCALD in colorectal cancer

**DOI:** 10.3892/ijo.2025.5768

**Published:** 2025-06-25

**Authors:** Shengli Pan, Yingying Deng, Jun Fu, Yuhao Zhang, Zhijin Zhang, Xianju Qin

Int J Oncol 60: 14, 2022; DOI: 10.3892/ijo.2022.5304

Subsequently to the publication of the above article, an interested reader drew to the authors' attention that certain of the tumor images shown in Fig. 7B on p. 13 had already appeared in different form in Fig. 2E in a previously published paper in the journal *Frontiers in Cell and Developmental Biology* written by different authors at different research institutes.

Owing to the fact that the contentious data in the above article had already been published prior to its submission to *International Journal of Oncology*, the Editor has decided that this paper should be retracted from the Journal. The authors were asked for an explanation to account for these concerns, but the Editorial Office did not receive a reply. The Editor apologizes to the readership for any inconvenience caused.

